# Evaluation of medical students’ awareness regarding acute rheumatic fever

**DOI:** 10.1371/journal.pone.0354855

**Published:** 2026-08-17

**Authors:** Ayben Leblebici, Alaa Almassri, Nazikgül Berdiyeva, Motaz Aref Baggash Al-Hamati, Birsen Uçar

**Affiliations:** 1 Department of Pediatrics, Faculty of Medicine, Eskisehir Osmangazi University, Eskisehir, Turkey; 2 Faculty of Medicine, Eskisehir Osmangazi University, Eskisehir, Turkey; 3 Department of Pediatric Cardiology, Faculty of Medicine, Eskisehir Osmangazi University, Eskisehir, Turkey; Bilawal Medical College, Liaquat University of Medical and Health Sciences, PAKISTAN

## Abstract

Acute rheumatic fever (ARF) is an autoimmune disease that develops as a complication of group A beta-hemolytic streptococcal infections and may lead to rheumatic heart disease, particularly in children and young adults in developing countries. With an annual incidence of approximately 10–20 per 100,000, Turkey is considered a moderate-to-high risk country. Early diagnosis, treatment, and prophylaxis are crucial in the prevention of ARF and rheumatic heart disease. This study aimed to evaluate the knowledge level of first-year medical students (in the basic medical education phase) and sixth-year students (who had completed their clinical clerkships) at Eskisehir Osmangazi University Faculty of Medicine during the 2019–2020 academic year, in accordance with the American Heart Association guidelines. A total of 165 students (93 first-year and 72 sixth-year) participated in the online survey. The results demonstrated that the knowledge level of sixth-year students was significantly higher compared to first-year students (p < 0.05). These findings highlight the importance of incorporating education on preventable diseases such as ARF into medical curricula.

## Introduction

Acute rheumatic fever (ARF) is an inflammatory disorder initiated by an autoimmune response that develops following inadequately treated group A beta-hemolytic streptococcal (GABHS) infections. It can involve multiple organs such as the heart, joints, brain, and skin, particularly in children, and may progress to rheumatic heart disease. The 2015 guidelines of the American Heart Association (AHA) emphasized the use of the Modified Jones criteria for the diagnosis of ARF and recommended benzathine penicillin G for secondary prophylaxis [1]. Subsequent AHA documents continued to support the clinical applicability of the revised Jones criteria [2].

The Centers for Disease Control and Prevention (CDC), in its most recent clinical guidance on ARF, updated the recommendations on the use of antibiotics such as benzathine penicillin G, emphasizing that early diagnosis and education are of critical importance in preventing the disease [3]. With an annual incidence estimated at 10–20 per 100,000, Turkey is classified as a moderate-to-high risk country. Rheumatic heart disease, the most severe complication of ARF, has been reported to have a prevalence of approximately 317 per 100,000 among children aged 5–14 years, and 661 per 100,000 nationwide [4]. The present study aimed to compare the awareness of ARF among medical students at the beginning of their training and those in their final year in Turkey.

## Materials and methods

This cross-sectional study was conducted at the Faculty of Medicine, Eskisehir Osmangazi University, during the 20.01.2020–1.06.2020 academic year. At that time, there were 284 students enrolled in the first year and 205 students in the sixth year. Ethical approval for the study was obtained from the Ethics Committee of Eskisehir Osmangazi University Faculty of Medicine on January 14, 2020, with decision number 04. Informed consent was obtained verbally from all participants prior to participation. Participants were informed about the study aims, voluntary participation, data anonymity, and their right to withdraw. Verbal consent was obtained in the presence of two witnesses, and only participants who provided consent were given access to the online questionnaire. No personal identifiers were collected. The study was carried out in accordance with the Declaration of Helsinki.

In our curriculum, students receive instruction on ARF in the third year covering pathology, pathogenesis, and clinical features, and in the fourth year focusing on clinical presentation, diagnosis, treatment, and prophylaxis. The study sample consisted of first-year students (n = 93) and sixth-year students (n = 72) who voluntarily agreed to participate in the survey. The online questionnaire administered to the volunteer students consisted of 14 items. The first three questions addressed demographic characteristics (gender, age, and year of study), while the subsequent multiple-choice items inquired about ARF findings, diagnosis, treatment, and prophylaxis in accordance with the AHA guidelines (e.g., signs of GABHS pharyngitis, Jones criteria, and duration of secondary prophylaxis). The questionnaire was designed to allow students to select more than one option where applicable. The questions included in the survey are presented in Table 1.

**Table 1:** Questions on Acute Rheumatic Fever Administered to First- and Sixth-Year Medical Students.

1	Sex
**2**	Age
**3**	Grade (First-year / Sixth-year medical student)
**4**	What are the clinical findings of group A beta-hemolytic streptococcal pharyngitis?
**5**	Which drug is the treatment of choice for group A beta-hemolytic streptococcal pharyngitis?
**6**	What is the underlying cause of acute rheumatic fever?
**7**	Which organs may be affected by acute rheumatic fever?
**8**	According to which criteria is the diagnosis of acute rheumatic fever established?
**9**	Which criteria are sufficient to make a diagnosis of acute rheumatic fever?
**10**	Which treatment is used in acute rheumatic fever?
**11**	What is the first-choice drug for secondary prophylaxis of acute rheumatic fever?
**12**	In a patient with penicillin allergy, which drug should be preferred for secondary prophylaxis of acute rheumatic fever?
**13**	If carditis is absent, for how many years should secondary prophylaxis of acute rheumatic fever be administered?
**14**	If carditis is present, for how many years should secondary prophylaxis of acute rheumatic fever be administered?

The number and proportion of correct responses were analyzed using cross-tabulation to assess the relationship between two categorical variables. The variables were defined as grade level (first-year, sixth-year) and response status (correct, incorrect). For each question, the proportions of correct and incorrect answers were compared according to grade level.

All analyses were performed using IBM SPSS Statistics for Windows, Version 21.0 (IBM Corp., Armonk, NY, USA). Continuous variables were expressed as mean ± standard deviation (SD). Categorical variables were analyzed using Pearson’s chi-square test, the continuity-corrected chi-square test, and Fisher’s exact test, as appropriate. Categorical data were presented as frequencies and percentages (n%). A p value < 0.05 was considered statistically significant.

## Results

The mean age of the participants was 19 ± 2 years in the first-year group and 24 ± 2 years in the sixth-year group, with 60% being female. While the first-year students were in the basic medical education phase, the sixth-year students had completed their clinical rotations and were working as interns. The responses of the students to the questionnaire items are presented in tables (Tables 2–12).

**Table 2:** Knowledge level of students regarding the clinical findings of group A beta-hemolytic streptococcal pharyngitis (Question 4).

Options	First-year (n = 93)	Sixth-year (n = 72)	p
Sore throat	8 (8.6%)	61 (84.7%)	
Exudates on pharynx and tonsils	3 (3.2%)	57 (79.1%)	
Anterior cervical lymphadenitis	3 (3.2%)	51 (70.8%)	
Hypertrophic tonsils	1 (1.1%)	54 (75.0%)	
I don’t know	83 (89.2%)	0 (0%)	
Correct response*	0 (0%)	41 (56.9%)	<0.001

*The number and percentage of students who selected all of the following correct findings: sore throat, exudates on pharynx and tonsils, anterior cervical lymphadenitis, and hypertrophic tonsils.

**Table 3:** Knowledge level of students regarding the treatment of group A beta-hemolytic streptococcal pharyngitis (Question 5).

Options	First-year (n = 93)	Sixth-year (n = 72)	p
Oral penicillin	2 (2.2%)	27 (37.5%)	
Oral amoxicillin	1 (1.1%)	10 (13.8%)	
Azithromycin	2 (2.2%)	0 (0%)	
Single-dose benzathine penicillin G (intramuscular)	2 (2.2%)	48 (66.6%)	
Procaine penicillin (intramuscular)	0 (0%)	9 (12.5%)	
Trimethoprim-sulfamethoxazole	0 (0%)	1 (1.4%)	
Cephalosporin	0 (0%)	4 (5.6%)	
I don’t know	86 (92.4%)	3 (4.2%)	
Correct response*	6 (6.5%)	64 (88.9%)	<0.001

*The number and percentage of students who selected at least one of the following correct options: oral penicillin, oral amoxicillin, azithromycin, single-dose benzathine penicillin G, or procaine penicillin.

**Table 4:** Knowledge level of students regarding the etiology of acute rheumatic fever (Question 6).

Options	First-year (n = 93)	Sixth-year (n = 72)	p
Cold weather	10 (10.7%)	1 (1.4%)	
Joint infection	15 (16.1%)	7 (9.7%)	
Throat infection	15 (16.1%)	67 (93.0%)	
Skin infection	1 (1.1%)	21 (29.1%)	
I don’t know	56 (60.2%)	2 (2.8%)	
Correct response*	11 (11.8%)	43 (59.7%)	<0.001

*Number and percentage of students who selected “throat infection” as the correct option.

**Table 5:** Knowledge level of students regarding the organs affected by acute rheumatic fever (Question 7).

Options	First-year (n = 93)	Sixth-year (n = 72)	p
Kidneys	19 (20.4%)	41 (56.9%)	
Heart	24 (25.8%)	71 (98.6%)	
Liver	12 (12.9%)	6 (8.3%)	
Skin	14 (15.0%)	43 (59.7%)	
Joints	23 (24.7%)	63 (87.5%)	
Brain	16 (17.2%)	19 (26.3%)	
I don’t know	55 (59.1%)	0 (0%)	
Correct response*	0 (0%)	6 (8.3%)	0.006

*Number and percentage of students who selected all of the following correct options: heart, skin, joints, and brain.

**Table 6:** Knowledge level of students regarding the diagnostic criteria for acute rheumatic fever (Question 8).

Options	First-year (n = 93)	Sixth-year (n = 72)	p
Modified Jones criteria	3 (3.2%)	63 (87.5%)	
Schwartz criteria	2 (2.2%)	1 (1.4%)	
Duke criteria	2 (2.2%)	5 (6.9%)	
I don’t know	86 (92.4%)	3 (4.2%)	
Correct response*	3 (3.2%)	63 (87.5%)	<0.001

*Number and percentage of students who selected “Modified Jones criteria” as the correct option.

**Table 7:** Knowledge level of students regarding the criteria required for the diagnosis of acute rheumatic fever (Question 9).

Options	First-year (n = 93)	Sixth-year (n = 72)	p
One major criterion	0 (0%)	9 (12.5%)	
Two minor criteria	2 (2.2%)	9 (12.5%)	
One minor criterion plus evidence of prior streptococcal infection	1 (1.1%)	3 (4.2%)	
Two major criteria plus evidence of prior streptococcal infection	4 (4.3%)	49 (68.0%)	
One major and two minor criteria plus evidence of prior streptococcal infection	1 (1.1%)	58 (80.5%)	
Sydenham’s chorea	1 (1.1%)	42 (58.3%)	
I don’t know	84 (90.3%)	2 (2.8%)	
Correct response*	0 (0%)	38 (52.7%)	<0.001

*Number and percentage of students who selected all of the following correct options: two major criteria plus evidence of prior streptococcal infection, one major and two minor criteria plus evidence of prior streptococcal infection, and Sydenham’s chorea.

**Table 8:** Knowledge level of students regarding the drugs used in the treatment of acute rheumatic fever (Question 10).

Options	First-year (n = 93)	Sixth-year (n = 72)	p
Aspirin	6 (6.5%)	36 (50.0%)	
Prednisolone	4 (4.3%)	34 (47.2%)	
Antibiotic	11 (11.8%)	44 (61.1%)	
I don’t know	73 (78.4%)	4 (5.6%)	
Correct response*	0 (0%)	6 (8.3%)	0.006

*Number and percentage of students who selected both aspirin and prednisolone.

**Table 9:** Knowledge level of students regarding the first-choice drug for secondary prophylaxis of acute rheumatic fever (Question 11).

Options	First-year (n = 93)	Sixth-year (n = 72)	p
Benzathine penicillin G	3 (3.2%)	44 (61.1%)	
Erythromycin	2 (2.2%)	9 (12.5%)	
Oral penicillin	1 (1.1%)	9 (12.5%)	
Azithromycin	2 (2.2%)	2 (2.8%)	
Cephalosporin	0 (0%)	5 (6.9%)	
Aminoglycosides	0 (0%)	1 (1.4%)	
I don’t know	85 (91.3%)	13 (18.0%)	
Correct response*	3 (3.2%)	36 (50.0%)	<0.001

*Number and percentage of students who selected “benzathine penicillin G” as the correct option.

**Table 10:** Knowledge level of students regarding the first-choice drug for secondary prophylaxis of acute rheumatic fever in patients with penicillin allergy (Question 12).

Options	First-year (n = 93)	Sixth-year (n = 72)	p
Erythromycin	5 (5.4%)	50 (69.4%)	
Azithromycin	1 (1.1%)	11 (15.2%)	
Clarithromycin	2 (2.2%)	16 (22.2%)	
Sulfonamide	0 (0%)	1 (1.4%)	
I don’t know	85 (91.3%)	10 (13.8%)	
Correct response*	5 (5.4%)	40 (55.6%)	<0.001

*Number and percentage of students who selected “erythromycin” as the correct option.

**Table 11:** Knowledge level of students regarding the duration of secondary prophylaxis for acute rheumatic fever in the absence of carditis (Question 13).

Options	First-year (n = 93)	Sixth-year (n = 72)	p
5 years	0 (0%)	22 (30.5%)	
10 years	2 (2.2%)	13 (18.0%)	
Until 21 years of age	1 (1.1%)	39 (54.1%)	
Until 25 years of age	0 (0%)	3 (4.2%)	
Lifelong	0 (0%)	0 (0%)	
I don’t know	90 (96.8%)	10 (13.8%)	
Correct response*	0 (0%)	9 (12.5%)	<0.001

*Number and percentage of students who selected both “5 years” and “until 21 years of age” as the correct options.

**Table 12:** Knowledge level of students regarding the duration of secondary prophylaxis for acute rheumatic fever in the presence of carditis (Question 14).

Options	First-year (n = 93)	Sixth-year (n = 72)	p
5 years	0 (0%)	2 (2.8%)	
10 years	3 (3.2%)	13 (18.0%)	
Until 21 years of age	0 (0%)	13 (18.0%)	
Until 25 years of age	0 (0%)	2 (2.8%)	
Lifelong	4 (4.3%)	41 (56.9%)	
I don’t know	86 (92.4%)	7 (9.7%)	
Correct response*	0 (0%)	1 (1.4%)	0.436

*Number and percentage of students who selected both “10 years” and “until 25 years of age” as the correct options.

The knowledge level regarding the findings of GABHS pharyngitis was found to be significantly higher among sixth-year students compared to first-year students.

The knowledge level of students regarding the treatment of GABHS pharyngitis was markedly higher in the sixth year. Among the options, sixth-year students most frequently selected single-dose benzathine penicillin G.

The knowledge level regarding the etiology of acute rheumatic fever was found to be statistically significantly higher among sixth-year students compared to first-year students.

In the question regarding the organs affected by acute rheumatic fever, the proportion of sixth-year students selecting heart, skin, and joints was markedly higher compared to first-year students. Moreover, the number of sixth-year students who selected all correct options (heart, skin, joints, and brain) was statistically significantly greater than that of first-year students.

The knowledge level of sixth-year students regarding the diagnostic criteria for acute rheumatic fever was statistically significantly higher compared to that of first-year students.

The knowledge level regarding the criteria used to establish the diagnosis of acute rheumatic fever was found to be statistically significantly higher among sixth-year students compared to first-year students.

The proportion of sixth-year students who selected aspirin and prednisolone as treatment options for acute rheumatic fever was statistically significantly higher compared to first-year students. Although a considerable proportion of students selected “antibiotic,” this response was not accepted as correct since antibiotics are used for prophylaxis rather than for the treatment of ARF.

The knowledge level regarding the first-choice drug for secondary prophylaxis of acute rheumatic fever was found to be statistically significantly higher among sixth-year students compared to first-year students.

The knowledge level regarding the first-choice drug for secondary prophylaxis of acute rheumatic fever in patients with penicillin allergy was found to be statistically significantly higher among sixth-year students compared to first-year students.

The knowledge level regarding the duration of secondary prophylaxis for ARF in the absence of carditis was found to be statistically significantly higher among sixth-year students compared to first-year students.

No statistically significant difference was found between first- and sixth-year students regarding their knowledge of the duration of secondary prophylaxis for ARF in the presence of carditis. However, the “lifelong” option was most frequently selected by sixth-year students.

The mean overall correct response rate was 2.74% among first-year students and 43.81% among sixth-year students. In first-year students, the highest correct response rates were observed for questions on the etiology of ARF (11.8%) and the treatment of GABHS pharyngitis (6.5%), while in several questions (4, 7, 10, 13, and 14) the correct response rate was 0%. Among sixth-year students, the highest correct response rate (88.8%) was noted for the question regarding the treatment of GABHS pharyngitis, whereas the lowest correct response rate (1.4%) was observed for the question on the duration of secondary prophylaxis for ARF in the absence of carditis. The largest differences between the responses of first- and sixth-year students were seen in clinically oriented questions, such as the findings and treatment of GABHS pharyngitis and the diagnostic criteria of ARF.

## Discussion

This pilot study evaluated awareness of acute rheumatic fever (ARF) among medical students at the beginning and at the end of undergraduate medical education. The results demonstrated that the knowledge level of sixth-year students who had completed clinical training was significantly higher than that of first-year students. The finding that the correct response rate was 0% for many questions among first-year students indicates a substantial lack of baseline knowledge regarding ARF. Although sixth-year students achieved higher correct response rates across all questions compared to first-year students, their overall correct response rate remaining at 31.44% is noteworthy. Despite having completed clinical training, overall awareness among sixth-year students was lower than expected, suggesting a potentially important gap in medical education.

Acute rheumatic fever remains a preventable disease that continues to pose a significant public health burden, particularly in low- and middle-income countries and in regions with moderate to high disease prevalence, including Turkey [4–7]. In this context, ensuring adequate awareness among future physicians is critical for early diagnosis, appropriate treatment, and effective secondary prevention.

The comparison between first- and sixth-year students was deliberately designed to examine changes in ARF awareness throughout the course of medical education. Although the differences between the two groups were statistically significant, the absolute level of correct responses among sixth-year students was still insufficient. Similar findings have been reported in previous studies conducted among medical students and healthcare professionals, demonstrating inadequate knowledge of ARF despite advanced education and clinical experience [8,9]. These findings suggest that progression through medical education alone may not be sufficient to ensure adequate awareness of ARF.

An important observation of this study is that sixth-year students showed particularly low correct response rates for clinically critical topics, such as the recommended duration of secondary prophylaxis for ARF. Current international guidelines clearly emphasize the importance of appropriate and sustained secondary prophylaxis to prevent disease recurrence and long-term cardiac complications [10]. Insufficient knowledge in these areas may therefore have direct clinical implications and adversely affect patient outcomes.

The limited level of knowledge regarding ARF observed among first-year medical students is an expected finding, given their early stage of medical education. Rather than reflecting a deficiency, this finding underscores the importance of a structured and progressive medical curriculum. However, the persistence of low awareness even among final-year students suggests that current undergraduate curricula may not place sufficient emphasis on ARF and its prevention strategies, despite the continued clinical relevance of the disease. This observation is consistent with previous studies. For example, a study conducted in Saudi Arabia reported low awareness of ARF among the general population, while another study from Egypt involving 629 physicians working in preventive healthcare settings reported a moderate level of knowledge [11]. Studies specifically evaluating the knowledge level of medical students on this topic remain limited. In Sudan, a study conducted among pediatric physicians—including interns in the final stage of medical education, medical residents, and postgraduate trainees—assessed knowledge related to the prevention of ARF and rheumatic heart disease. In that study, physicians initially demonstrated a low level of knowledge regarding the diagnosis and treatment of group A beta-hemolytic streptococcal (GABHS) pharyngitis (38%); however, this rate increased to 93% following educational interventions such as seminars and the distribution of treatment protocols. This finding indicates that even individuals in the final stages of medical training may lack sufficient knowledge [12]. Similarly, a study from Cameroon involving 509 final-year medical students reported that 58.2% of participants had a moderate level of knowledge regarding rheumatic heart disease, and only one-third demonstrated above-average knowledge [13].

Encouragingly, previous studies have demonstrated that targeted educational interventions can significantly improve awareness and knowledge of ARF and rheumatic heart disease among healthcare trainees [9]. This supports the notion that the deficiencies identified in our study are modifiable and highlights the potential value of integrating guideline-based educational strategies focused on both preclinical and clinical training periods.

This study has several limitations. The relatively small sample size and single-center design limit the generalizability of the findings and preclude definitive conclusions. Participation was voluntary, which may have introduced selection bias. In addition, although the questionnaire was developed based on relevant literature and expert opinion to ensure content validity, formal validity and reliability analyses were not performed. Therefore, the results should be interpreted as exploratory and hypothesis-generating. In future research, the same cohort of students surveyed in the first year could be reassessed longitudinally in their sixth year.

In conclusion, this study confirms that the clinical phases of medical education enhance knowledge regarding ARF. However, since even sixth-year students demonstrated relatively low awareness of ARF, it is considered essential—particularly in moderate-to-high risk regions such as our country—to provide physicians with regular educational programs on ARF and rheumatic heart disease, and to integrate the AHA guidelines into medical curricula. Such measures are expected to enable future physicians to manage this preventable disease more effectively.

## Supporting information

S1 Data Supporting Information S1 Data-PlosOne. (XLSX)
